# A systematic review of the effect of pre-test rest duration on toe and ankle systolic blood pressure measurements

**DOI:** 10.1186/1756-0500-7-213

**Published:** 2014-04-05

**Authors:** Sean Sadler, Vivienne Chuter, Fiona Hawke

**Affiliations:** 1 The University of Newcastle, Ourimbah, Australia

**Keywords:** Rest time, Ankle brachial index, Toe brachial index, Peripheral arterial disease

## Abstract

**Background:**

Measurement of toe and ankle blood pressure is commonly used to evaluate peripheral vascular status, yet the pre-test rest period is inconsistent in published studies and among practitioners, and could affect results. The aim of this systematic review is to evaluate all research that has investigated the effect of different periods of pre-test rest on toe and ankle systolic blood pressure.

**Methods:**

The following databases were searched up to April 2012: Medline (from 1946), EMBASE (from 1947), CINAHL (from 1937), and Cochrane Central Register of Controlled Trials (CENTRAL) (from 1800). No language or publication restrictions were applied. Eighty-eight content experts and researchers in the field were contacted by email to assist in the identification of published, unpublished, and ongoing studies. Studies evaluating the effect of two or more pre-test rest durations on toe or ankle systolic blood pressure were eligible for inclusion. No restrictions were placed on participant characteristics or the method of blood pressure measurement. Outcomes included toe or ankle systolic blood pressure and adverse effects. Abstracts identified from the search terms were independently assessed by two reviewers for potential inclusion.

**Results:**

1658 abstracts were identified by electronic searching. Of the 88 content experts and researchers in the field contacted by email a total of 33 replied and identified five potentially relevant studies. No studies were eligible for inclusion.

**Conclusions:**

There is no evidence of the effect of different periods of pre-test rest duration on toe and ankle systolic blood pressure measurements. Rigorous trials evaluating the effect of different durations of pre-test rest are required to direct clinical practice and research.

## Background

Duration of pre-test rest time for blood pressure measurement varies markedly in the literature, ranging from 5 minutes [1-3] to 30 minutes [4-8]. This range of pre-test rest times reflects inconsistencies between clinical guidelines. Additionally, current National Institute for Health and Clinical Excellence (NICE) guidelines [9] do not provide definitive guidance on optimum pre-test rest duration for toe, ankle, or brachial systolic blood pressure measurements. Brachial systolic blood pressure, when measured in a supine [10] or seated [11] position continues to fall throughout the first 10 minutes of pre-test resting. Measurement of blood pressure before resting systolic blood pressure is achieved would cause a falsely elevated reading. Similar changes may also occur in lower limb blood pressure and may affect the measurements routinely used in lower limb vascular assessment.

The length of pre-test rest time is not only an important factor for stabilisation of blood pressure, reliability of the measurement, and accurate diagnosis of peripheral arterial disease (PAD), but also for clinical efficiency. Mohler III and colleagues [12] surveyed 897 clinicians and found that the principal factor limiting office utilisation of Ankle Brachial Indices (ABIs) was time restraints. Similarly, Chen, Lawford, Shah, Pham and Bower [13] found in a cross sectional survey of 92 Western Australian Podiatrists that time restrictions were the underlying reason for clinicians’ infrequent use of ABIs. The lack of clinical utilisation of vascular assessments is understandable when considering some studies suggest clinicians should wait 30 minutes before performing an ABI, which then takes an additional 5 minutes [14]. Guidelines that provide clinicians with an efficient protocol for performing non-invasive vascular assessments could improve their usage.

This systematic review aims to evaluate all research that has investigated the effect of different periods of pre-test rest time on toe and ankle systolic blood pressure measurements in humans. Determination of the shortest duration of pre-test rest that produces both valid and reliable results may improve clinical efficiency, increase vascular assessment utilisation, and guide future research. This systematic review has been published as conference proceedings [15].

## Methods

### Inclusion and exclusion criteria

All studies measuring toe or ankle systolic blood pressure in any person after two or more periods of pre-test rest were eligible for inclusion. We planned to include all methods of blood pressure measurement, including automated or manually operated devices, and blood pressures measured from any toe and from either the dorsalis pedis or posterior tibial arteries. We excluded exercise stress tests, post-occlusive hyperaemia tests, and studies of toe or ankle systolic blood pressure measurement that used only one pre-test rest period.

### Outcomes

The primary outcome was change in toe and ankle systolic blood pressure over time and the secondary outcome was adverse events associated with taking the measurements.

### Search strategy

The following databases were searched up to April 2012:

1) MEDLINE (from 1946) (Additional file 1 - MEDLINE search strategy);

2) EMBASE (from 1947) (Additional file 2 - EMBASE search strategy);

3) CINAHL (from 1937) (Additional file 3 - CINAHL search strategy); and

4) Cochrane Central Register of Controlled Trials (CENTRAL) (from 1800) (Additional file 4 - CENTRAL search strategy).

Studies identified from the search terms were not subject to language or publication restrictions.

### Other sources

Eighty-eight content experts and researchers in the field were contacted via email and asked to identify potentially relevant studies.

### Data collection and analysis

Two review authors (SS and FH) independently assessed titles and abstracts (where available) of all studies identified by the search. No disagreements occurred while screening for inclusion so no arbitration by a third reviewer (VC) was needed. Data extraction was planned to be conducted by one reviewer (SS) using a pilot-tested form and to be cross-checked by a second reviewer (FH).

As no gold standard appraisal tool exists for studies investigating measurement agreement, we planned to assess risk of bias of included studies using the QAREL tool [16] and the completeness of reporting using the STROBE tool [17].

## Results

Electronic searches retrieved a total of 1658 citations (555 from MEDLINE; 781 from EMBASE; 246 from CINAHL; and 76 from CENTRAL). After screening all identified studies at a title and abstract level, none was eligible for inclusion therefore no full text versions of studies were required. Thirty three content experts and researchers in the field (Additional file 5 - Experts contacted) replied to our email (Additional file 6 - Email sent to experts) and identified five potentially relevant studies, none of which was eligible for inclusion. Two ongoing studies by Chuter and Casey (2012a) (unpublished observations) and Chuter and Casey (2012b) (unpublished observations) were identified but no data were available for inclusion in this review (Additional file 7 - PRIMSA flow diagram).

Studies were excluded because the researchers investigated the effects of post-occlusive reactive hyperaemia on rest time; the effects of exercise stress tests on rest time; and blood pressures were measured after one period of rest time.

## Discussion

No study has evaluated the effect of different durations of pre-test rest on toe or ankle systolic blood pressure measurements.

Although there have been no studies investigating the effect of different durations of pre-test rest time on toe and ankle systolic blood pressure, there is some evidence for the effects of different pre-test rest durations on brachial systolic blood pressure. Based on the studies investigating the effects of different periods of pre-test rest duration on brachial systolic blood pressure, it appears that the hydrostatic effects of gravity on blood pressure are reduced after approximately 10 minutes of either supine [10] or chair-seated rest [11] and no further reduction is gained by a longer rest period [18].

### Limitations and overall completeness

Due to the lack of research evidence, this systematic review cannot provide a clear recommendation for the ideal pre-test rest duration for measuring toe or ankle systolic blood pressures. The overall completeness of this review was strengthened by thoroughly searching electronic databases, and communicating extensively, in English and non-English language, with dozens of national and international content experts and researchers in the field. This is the first systematic review to investigate the effects of pre-test rest duration on toe and ankle systolic blood pressure measurements, therefore no agreements or disagreements with other studies or reviews occurred.

More research is also needed to determine the minimum number of participants needed to detect clinically important differences between periods of pre-test rest duration when measuring toe and ankle systolic blood pressure.

## Conclusion

There is no evidence to determine if different periods of pre-test rest duration affect toe or ankle systolic blood pressure. This review highlights the urgent need for rigorously designed research evaluating the effect of pre-test rest duration on toe or ankle systolic blood pressures when measured after at least two periods of pre-test rest. Outcomes should include toe or ankle systolic blood pressure and adverse effects associated with taking the measurements.

## Abbreviations

ABI: Ankle brachial index; TBI: Toe brachial index; PAD: Peripheral arterial disease.

## Competing interests

The authors declare that they have no competing interests.

## Authors’ contributions

SS was responsible for retrieving studies, contacting content experts and researchers in the field, and writing the review. FH was responsible for conceiving the review and providing detailed comments on the review. SS and FH were responsible for developing the search terms, screening studies, and interpreting results. VC was responsible for providing detailed comments on the review and redrafting of the introduction and discussion. All authors read and approved the final manuscript.

## Supplementary Material

Additional file 1 MEDLINE search strategy; key words used to search the MEDLINE database. Click here for file

Additional file 2 EMBASE search strategy; key words used to search the EMBASE database. Click here for file

Additional file 3 CINAHL search strategy; key words used to search the CINAHL database. Click here for file

Additional file 4 CENTRAL search strategy; key words used to search the Cochrane Central Register of Controlled Trials (CENTRAL). Click here for file

Additional file 5 Experts contacted; the list of content experts and researchers in the field contacted and studies suggested for potential inclusion. Click here for file

Additional file 6 Email sent to experts; the email text sent to content experts and researchers in the field requesting published, unpublished, or ongoing studies. Click here for file

Additional file 7 PRIMSA flow diagram; PRIMSA flow diagram of retrieved, screened, included, and excluded articles. Click here for file
