# Supplementary material for: A systematic review of the effect of pre-test rest duration on toe and ankle systolic blood pressure measurements
Source: BMC Res Notes. 2014 Apr 5;7:213. doi: 10.1186/1756-0500-7-213 (PMC4234995; doi:10.1186/1756-0500-7-213)
Supplement: Additional file 1 — MEDLINE search strategy; key words used to search the MEDLINE database. [file 1756-0500-7-213-S1.pdf]

Additional file 1: MEDLINE search strategy

1.	ankle adj2 blood adj2 pressure$.tw.
2.	toe adj2 blood adj2 pressure$.tw.
3.	hallux adj2 blood adj2 pressure$.tw.
4.	big adj2 toe adj2 blood adj2 pressure$.tw.
5.	ankle adj2 pressure$.tw.
6.	toe adj2 pressure$.tw.
7.	hallux adj2 pressure$.tw.
8.	big adj2 toe adj2 pressure$.tw.
9.	toe adj2 brachial adj2 ind$.tw.
10.	ankle adj2 brachial adj2 ind$.tw.
11.	ankle adj2 arm adj2 ind$.tw.
12.	toe adj2 brachial adj2 pressure$ adj2 ind$.tw.
13.	ankle adj2 brachial adj2 pressure$ adj2 ind$.tw.
14.	ankle adj2 arm adj2 pressure$ adj2 ind$.tw.
15.	1 or 2 or 3 or 4 or 5 or 6 or 7 or 8 or 9 or 10 or 11 or 12 or 13 or 14
16.	rest adj2 time$.tw.
17.	rest adj2 interval$.tw.
18.	minute$.tw.
19.	hour$.tw.
20.	min.tw.
21.	hr.tw.
22.	16 or 17 or 18 or 19 or 20 or 21
23.	15 and 22
24.	exp animals/ not humans.sh.
25.	23 not 24
