# Supplementary material for: A systematic review of the effect of pre-test rest duration on toe and ankle systolic blood pressure measurements
Source: BMC Res Notes. 2014 Apr 5;7:213. doi: 10.1186/1756-0500-7-213 (PMC4234995; doi:10.1186/1756-0500-7-213)
Supplement: Additional file 3 — CINAHL search strategy; key words used to search the CINAHL database. [file 1756-0500-7-213-S3.pdf]

Additional file 3: CINAHL search strategy

1.	ankle N2 blood N2 pressure*.tx.
2.	toe N2 blood N2 pressure*.tx
3.	hallux N2 blood N2 pressure*.tx.
4.	big N2 toe N2 blood N2 pressure*.tx.
5.	ankle N2 pressure*.tx.
6.	toe N2 pressure*.tx.
7.	hallux N2 pressure*.tx.
8.	big N2 toe N2 pressure*.tx.
9.	toe N2 brachial N2 ind*.tx
10.	ankle N2 brachial N2 ind*.tx.
11.	ankle N2 arm N2 ind*.tx.
12.	toe N2 brachial N2 pressure* N2 ind*.tx
13.	ankle N2 brachial N2 pressure* N2 ind*.tx
14.	ankle N2 arm N2 pressure* N2 ind*.tx
15.	S1 or S2 or S3 or S4 or S5 or S6 or S7 or S8 or S9 or S10 or S11 or S12 or S13 or S14
16.	rest N2 time*.tx.
17.	rest N2 interval*.tx
18.	minute*.tx.
19.	hour*.tx.
20.	TX min
21.	TX hr
22.	S16 or S17 or S18 or S19 or S20 or S21
23.	S15 and S22
