# Supplementary material for: A systematic review of the effect of pre-test rest duration on toe and ankle systolic blood pressure measurements
Source: BMC Res Notes. 2014 Apr 5;7:213. doi: 10.1186/1756-0500-7-213 (PMC4234995; doi:10.1186/1756-0500-7-213)
Supplement: Additional file 4 — CENTRAL search strategy; key words used to search the Cochrane Central Register of Controlled Trials (CENTRAL). [file 1756-0500-7-213-S4.pdf]

Additional file 4: CENTRAL search strategy

1.	(ankle NEAR/2 blood NEAR/2 pressure* or toe NEAR/2 blood NEAR/2 pressure* or hallux NEAR/2 blood NEAR/2 pressure* or big NEAR/2 toe NEAR/2 blood NEAR/2 pressure* or ankle NEAR/2 pressure* or toe NEAR/2 pressure* or hallux NEAR/2 pressure* or big NEAR/2 toe NEAR/2 pressure* or toe NEAR/2 brachial NEAR/2 ind* or ankle NEAR/2 brachial NEAR/2 ind* or ankle NEAR/2 arm NEAR/2 ind* or toe NEAR/2 brachial NEAR/2 pressure* NEAR/2 ind* or ankle NEAR/2 brachial NEAR/2 pressure* NEAR/2 ind* or ankle NEAR/2 arm NEAR/2 pressure* NEAR/2 ind*) AND
2.	(rest NEAR/2 time* or rest NEAR/2 interval* or minute* or hour* or hr or min) NOT
3.	(exp animals not humans.sh.)
