# Supplementary material for: A systematic review of the effect of pre-test rest duration on toe and ankle systolic blood pressure measurements
Source: BMC Res Notes. 2014 Apr 5;7:213. doi: 10.1186/1756-0500-7-213 (PMC4234995; doi:10.1186/1756-0500-7-213)
Supplement: Additional file 5 — Experts contacted; the list of content experts and researchers in the field contacted and studies suggested for potential inclusion. [file 1756-0500-7-213-S5.pdf]

Additional file 5: Experts contacted

Name	Response
Dr Gordon Hendry (University of Western Sydney, Australia)	Cloete et al. 2009; Romanos et al 2010
Sylvia McAra	Pérez-Martin et al. 2010
Dr George Murley (La Trobe University, Australia)	Romanos et al. 2010
Dr Dirk Ubbink	de Graaff et al. 2001
Peter Roberts	Aboyans et al. 2003
Prof Alan Bryant (University of Western Australia)	No studies suggested
A/Prof Virginia Bower	No studies suggested
A/Prof Laurie Foley	No studies suggested
Michael Concannon (University of Huddersfield, England)	No studies suggested
Prof Roger Eston (University of South Australia)	No studies suggested
Dr Karl Landorf (La Trobe University, Australia)	No studies suggested
Prof John Venson (Samuel Merrit University, School of Podiatric Medicine, America)	No studies suggested
Dr Thomas DeLauro (New York College of Podiatric Medicine, America)	No studies suggested
University of Salford Manchester, England (general email)	No studies suggested
Plymouth University, England (general email)	No studies suggested
Craig Lambert (University of Johannesburg, South Africa)	No studies suggested
Fatima Cassim	No studies suggested
National Institute for Health and Clinical Excellence	No studies suggested
Dr Phyllis Bonham (Medical School of South Carolina, America)	No studies suggested
Lars Jødal	No studies suggested
Mary Romanos	No studies suggested
Prof Gerry Fowkes	No studies suggested
Dr Charles Andersen	No studies suggested
A/Prof Maria Svensson	No studies suggested
Prof Nicholaas Schaper	No studies suggested
Dr John Allen	No studies suggested
A/Prof Matthew Allison	No studies suggested
Dr Karel Bakker	No studies suggested
Dr Michael Jaff	No studies suggested
Prof Alan Hirsch	No studies suggested
Dr Crispian Oates	No studies suggested
Prof Michael Criqui	No studies suggested
Cliff Coleman	No studies suggested
A/Prof Andrew Crowther (Charles sturt University, Australia)	Out of office
Dr Thomas Rooke	Out of office
Adjunct Prof Maria Hunink	Out of office
Prof Keith Rome (Auckland University of Technology, New Zealand)	None

A/Prof Paul Tinley (Charles Sturt University, Australia)	None
Dr Paul Bennett (Queensland University of Technology, Australia)	None
Dr Jeffrey Page (Midwestern University, America)	None
Barry University, School of Podiatric Medicine, America (general email)	None
Des Moines University, America (general email)	None
Ohio College of Podiatric Medicine, America (general email)	None
Prof Christine Espinola-Klein	None
Dr Morimoto Satoshi	None
Dr Mo Al-Qaisi	None
Dr Pahlsson	None
Prof Takashi Ohta	None
Prof Andrew Boulton	None
Dr Javier Ena	None
Dr Brigitte Esoubet	None
Prof Jeffrey Olin	None
A/Prof Ogedegbe	None
Dr Andreas Patzak	None
Dr Jue Li	None
College of Podiatric Medicine at Western University of Health Sciences, America (general email)	None
Glasgow Caledonian University, Scotland (general email)	None
Cardiff Metropolitan University, Wales (general email)	None
Fiona Dickenson (University of Northampton, England)	None
Birmingham Metropolitan College, England (general email)	None
University of Brighton, England (general email)	None
Lynne Flynn (Queen Margaret University, Scotland)	None
University of Ulster, Ireland (general email)	None
Prof Neville Punchard (University of East London, England)	None
The Michener Institute, Canada (general email)	None
Dr Herman van Langen	None
Dr Antonia Perez-Martin	None
Dr Ruth Taylor-Piliae	None
Dr Dean Williams	None
Dr Magnus Löndahl	None
Dr Nader Khandanpour	None
Dr Louis.Potier	None
Dr Dimitri Aerden	None
Dr Victor Aboyans	None
Dr Seung Hwan Han	None
Dr Hirofumi Tanaka	None
Prof Lars Norgren	None
Prof Alan Sinclair	None

Dr Sanjay Misra	None
A/Prof Joshua Beckman	None
Belinda Brooks	Email failed
Cynthia Fleck	Email failed
Dr Knut Kroger	Email failed
Dr Jette Joensen	Email failed
Dr Jan Eriksson	Email failed
A/Prof Chi Yung-Wei	Email failed
Prof William Hiatt	Email failed
Prof Leonne Prompers	Email failed
