# Supplementary material for: A systematic review of the effect of pre-test rest duration on toe and ankle systolic blood pressure measurements
Source: BMC Res Notes. 2014 Apr 5;7:213. doi: 10.1186/1756-0500-7-213 (PMC4234995; doi:10.1186/1756-0500-7-213)
Supplement: Additional file 6 — Email sent to experts; the email text sent to content experts and researchers in the field requesting published, unpublished, or ongoing studies. [file 1756-0500-7-213-S6.pdf]

Additional file 6: Email sent to experts

Email header: Request for assistance: Systematic review of rest time on measurement of toe or ankle blood pressure

Email text:
Dear

My name is Sean Sadler. I am a new graduate Podiatrist and am studying towards a Bachelor of Health Science Honours at the University of Newcastle Australia, supervised by Mrs Fiona Blyton and Dr Vivienne Chuter. As an introduction to my thesis, I am conducting a systematic review of the effect of rest time on toe and ankle systolic blood pressure measurement. Electronic searching of MEDLINE, EMBASE, CINAHL and CENTRAL identified 1658 abstracts, none of which met our inclusion criteria.

We are now contacting experts in the field to uncover other potentially relevant studies. Do you know of any published, unpublished or ongoing studies that meet the below selection criteria?

Studies evaluating the level of agreement of toe or ankle systolic blood pressures when measured after at least two rest times. No restrictions were placed on the age of participants or the type of blood pressure device(s) used. Outcomes included blood pressure and adverse effects.

Studies evaluating the reliability or diagnostic accuracy of toe or ankle blood pressure measurements using ONE rest time were excluded. Studies evaluating the effects of exercise stress tests or post-occlusive hyperaemia tests on toe or ankle blood pressure were also excluded.

If you are aware of any studies that potentially meet these criteria, please contact me by phone (+61422768988) or email (sean.sadler@uon.edu.au) by Friday 4th May.

Kind regards,

Sean Sadler
Podiatrist
Honours Student
